# The Impact of COVID-19 on Physical Activity Patterns of Dental Students: A Multinational Survey

**DOI:** 10.3390/healthcare10112140

**Published:** 2022-10-27

**Authors:** Sameer Badri AL-Mhanna, Wan Syaheedah Wan Ghazali, Mahaneem Mohamed, Roshan Noor Mohamed, Mubashir Baig Mirza, Syed Nahid Basheer, Suraj Arora, Hafeez Abiola Afolabi, Yahkub Babatunde Mutalub, Mohammed Dauda Goni, Abdulrahman M. Sheikh

**Affiliations:** 1 Department of Physiology, School of Medical Sciences, Universiti Sains Malaysia, Kubang Kerian 16150, Kelantan, Malaysia; 2 Department of Pediatric Dentistry, Faculty of Dentistry, Taif University, P.O. Box 11099, Taif 21944, Saudi Arabia; 3 Department of Conservative Dental Sciences, College of Dentistry, Prince Sattam Bin Abdulaziz University, P.O. Box 173, Al-Kharj 11942, Saudi Arabia; 4 Division of Operative Dentistry, Department of Restorative Dental Sciences, College of Dentistry, Jazan University, Jazan 45142, Saudi Arabia; 5 Department of Restorative Dental Sciences, College of Dentistry, King Khalid University, P.O. Box 960, Abha 61421, Saudi Arabia; 6 Department of General Surgery, School of Medical Sciences, Universiti Sains Malaysia, Kubang Kerian 16150, Kelantan, Malaysia; 7 Department of Clinical Pharmacology, College of Medical Sciences, Abubakar Tafawa Balewa University, Bauchi 74027, Nigeria; 8 Faculty of Veterinary Medicine, Universiti Malaysia Kelantan, City Campus, Kota Bharu 16100, Kelantan, Malaysia; 9 Faculty of Health Science, Somali International University, Mogadishu, Somalia

**Keywords:** dental education, 2019 novel coronavirus diseases, facilitators, lifestyle, exercise, well-being

## Abstract

Background: The authorities of the world had to take extraordinary containment measures due to the coronavirus (COVID-19) pandemic spreading across the globe. The only way to stay active during the pandemic was at-home physical activity (PA). The current study evaluates how these preventative measures impacted the PA and well-being of students. Methods: This study is multicentral and was conducted in Malaysia, India, Cambodia, and Saudi Arabia; participants were recruited from four different regions to answer the online questionnaire provided via a link shared using their personal WhatsApp, Facebook Messenger, and Twitter social media. Results: The means of vigorous, moderate, and light PA (min/day) between the active and inactive groups were significantly different (*p* = 0.001, 0.007, and 0.001), respectively. In comparison with pre-COVID-19, the participants reported that it became more challenging to engage in regular exercise since the onset of social distance, associated with a lack of motivation followed by “less confidence”, “less enjoyment”, “less support, and fewer opportunities to engage in exercise”; moreover, it was “difficult to maintain close relationships” and “hard to voice their options on contentious matters” (*p* = 0.001). Public health measures affected the PA and well-being of active and inactive students; this demonstrates that health promotion strategies aimed at enhancing levels of PA in inactive students may be necessary to improve students’ well-being.

## 1. Introduction

In December 2019, a novel strain of coronavirus was identified as the cause of a health menace and many cases of pneumonia in China. The disease was named coronavirus disease (COVID-19) and declared a pandemic by the World Health Organization (WHO, Geneva, Switzerland) [1]. COVID-19 originated in China and spread into Iran, South Korea, Japan, and later into most of Europe and the United States [2]. By April 2020, the confirmed positive cases were more than 800,000 globally, with more than 40,000 mortalities [3]. Approaching the middle of 2020, COVID-19 had infected approximately 2.9 million individuals in over 200 countries with more than 200,000 mortalities [4]. Up until 25 January 2021, more than 99 million cases with 2.1 million deaths were reported worldwide [5].

In Malaysia, the first confirmed case was on 25 January 2020, followed by the first outbreak wave that started on 27 February 2020 [6,7]. On 16 March 2020, the Malaysian Prime Minister issued a nationwide lockdown called the Movement Control Order (MCO) [8]. This MCO included Cambodia, India, and Saudi Arabia.

In Cambodia, the first confirmed case was on 27 January 2020, and in mid-March 2020, community mitigation measures were established [9], for example, closure of learning institutions and night venues, as well as the cancellation of significant events and limits on in-country travel in April [10]. In India, the first case was reported in January 2020, and on 24 March 2020, a nationwide lockdown was enforced [11,12]. In Saudi Arabia, the first outbreak was announced on 3 March 2020. Saudi Arabia, like many other countries around the world, locked most public and commercial services and imposed state-wide population movement restrictions [13]. These enacted measures from the governments of the respective countries had a detrimental effect on the social and psychological well-being of citizens, especially students [14]. It was apparent from the restrictions on students that their physical activity (PA) behavior and well-being would be adversely affected [15], because the students were exposed to psychological fatigue and limited physical activities due to the restrictions following the cessation of all sports and educational activities, such as stadiums and recreation facilities [16,17]. The aftermath effect of these setbacks created an imbalance in the mental well-being, PA, motivated behavior status, and concentration amongst students [18,19]. Brooks and Webster [20] indicated that the COVID-19 pandemic and the quarantine had been noted to lead to adverse psychological effects such as anxiety, stress syndrome, anger, depression, social isolation, homesickness, and travel restrictions, which may influence students’ mental health.

Following these concerns, the health, safety, and well-being of all the students inside and outside the campuses became the priority of institution authorities, because the unforeseen COVID-19 consequences would affect the students’ physical well-being, study focus and mood, rise in sedentary behavior, and student productivities [21]. Therefore, students were encouraged to continue their indoor physical activities to keep fit and healthy and prevent the spread of COVID-19 by abiding by stipulated control and preventive measures already put in place by the government and institution authorities [22].

It is stated that exercise can reduce psychological disorders and improve health status and vital factors that may decrease the risk of severe respiratory distress syndrome [23]. Regular PA is intensely linked with fitness and well-being, and individuals who practice moderate exercises regularly are likely to experience less strain, sadness, and nervousness [24].

The COVID-19 pandemic psychologically impacted public health, older adults, patients, adolescents, and college students in most countries [25,26,27,28]. The literature revealed the impact of the COVID-19 pandemic on individuals’ risk of disease before and after the pandemic [29,30]. However, no reported study about the well-being and PA behavior of dental students in Malaysia, India, Cambodia, and Saudi Arabia exposed to the COVID-19 pandemic has been carried out to date.

Hence, the primary aim of this study is to describe the PA and well-being of dental students from Malaysia, India, Cambodia, and Saudi Arabia during the lockdown measures that were enacted by governments at the beginning of the COVID-19 pandemic. Furthermore, we determined the changes in PA engagement, barriers, and facilitators among the active and inactive students in relation to the situation prior to the lockdown. We also explored the differences in indoor and outdoor PA based on the students’ well-being.

## 2. Materials and Methods

### 2.1. Study Population and Sample

The present study is a cross-sectional study that used online surveys (validated questionnaire) [30] to describe students’ responses to (nature of PA, well-being, current feelings, barriers to PA, and PA behaviors) pre- and during-COVID-19 containment measures imposed by the governments. The questionnaire was in two sets: set-A was used to describe the students’ PA events before the pandemic, while the second part (set-B) describes the same students’ PA events during the pandemic. The target population included dental students from Malaysia, India, Cambodia, and Saudi Arabia. The sample size for this study was calculated via G*Power software version 3.1.9.2. Using a type I error of 0.05, power of 80%, group number of 2, and medium effect size 0.25 gave a maximum sample size of 128. A correlation between the repeated measurements of 0.5, and a non-sphericity correction ε of 1 was used in computation of the sample size. A total of 509 students (pre-pandemic: 98, during pandemic: 411) were included in the current study. All the recruited students filled out the consent form and voluntarily agreed to take part in the study. The participants answered the online questionnaire via a link shared using their personal WhatsApp, Facebook Messenger, and Twitter social media. The questionnaires employed in this study were coded to ensure participants’ confidentiality. Demographics, PA behavior, outdoor PA, and well-being factors were all included in the survey. Age, sex, and country were among the demographic characteristics. The study received institutional ethical approval from the ethics committee, Taif University HAO-02-T-105.

### 2.2. Physical Activity Behaviour

The PA measurement was evaluated during COVID-19 restrictions using the validated Godin Leisure Questionnaire (English version) to investigate the present rate of participant’s PA and to classify participants as “active” or “inactive” based on the amount of recorded vigorous, moderate, and light PA engagement [31]. Based on conventional PA recommendations for health-related benefits from the Godin Leisure Questionnaire, the cutoff for active was >150 min of moderate–vigorous PA per week, whereas the relevant cutoff for inactive was <149.9 min of moderate–vigorous PA per week [32]. To measure current PA behavior, additional PA questions were administered. This examined whether PA had changed (“same”, “more”, or “less”) since the restrictions commenced, the most common type of PA, the location of PA (indoors, outdoors, or both), and if the location had changed due to social distancing measures. Moreover, the participant’s response was divided into 3 Likert scales of more active, same, and less active to assess PA barriers and facilitators regarding potential advantages, pleasure, confidence, and preparation of PA behavior. Further questions were used to understand the possible effect of social distancing on challenges, support, and opportunities for participation in PA. The student’s well-being was assessed using the Mental Health Continuum (MHC-SF) [33].

### 2.3. Outdoor Physical Activity

Students reported the number of minutes they spent each day engaging in outdoor PA, the importance of the nature of the activity, and whether the practice took place in a natural environment. Moreover, additional items from the nature-relatedness scale were also included based on their influence on PA behavior [34]. The nature-relatedness scale provides questions regarding nature-relatedness to assess an individual’s emotional, cognitive, and physical relationship to the natural world. Each item is graded on a Likert scale of 1 (strongly disagree) to 5 (strongly agree), and then a measure of nature-relatedness is calculated. The nature-relatedness scale has been linked to behavior, environmental factors, and the frequency of time spent in nature. Participants were also asked to rate their level of connection to nature on a sliding scale from 1 to 10 [35].

### 2.4. Inclusion Criteria and Exclusion Criteria

All undergraduate dental students of the targeted institution who have access to the institution’s social media groups were included in the study. Postgraduates and dental auxiliary students were excluded from the study.

### 2.5. Statistical Analysis

The data analysis tools include demographic statistics to describe the variables and inferential statistics to show the relationship and possible significance of the associations. Independent *t*-tests and chi-square tests analyzed population variations around the sexes. Participants were classified into “inactive” and “active” groups to examine PA behavior, underlying barriers, and facilitators to health and wellness results. Comparative analysis was carried out using independent *t*-tests and one-way ANOVA. Participants were also grouped based on self-reported changes in PA behavior to investigate variations between the active and inactive categories and related inhibitors, facilitators, and well-being results. Thus, one-way ANOVA and Tukey post hoc tests were carried out to explore the differences among participants who became more active, stayed the same, or became less active because of COVID-19 restrictions. Finally, independent *t*-tests and one-way ANOVA were performed to determine discrepancies between active and inactive subjects and relevant outdoor PA and well-being measures. Both statistical analyses were measured using the program SPSS-25.0, and significance was set at *p* < 0.05.

## 3. Results

Participants’ sociodemographic data are shown in Table 1. A total of 411 respondents completed the questionnaire from four institutions, each from four different countries during the pandemic, and the results were compared with the pre-pandemic findings, including 98 respondents as the control group.

### 3.1. Physical Activity Engagement, Facilitators, Barriers, and Change in Type of Activities since COVID-19

Table 2 shows that the vigorous PA and light PA means in min/each day were significantly different (*p* < 0.001), respectively. Further, vigorous PA (day/week) was significantly different between the “active” and “inactive” groups (*p* < 0.001). There was a significant difference between inactive and active participants, and the behavior of PA changed since several restrictions on COVID-19 were put in place (*p* < 0.001). The results indicated that during COVID-19, the participants became increasingly less active, as 27.5% of the inactive students became “less active”, while 12.2% of active students became “less active”. Hence, due to the COVID-19 pandemic, most students became inactive since the restrictions were implemented. In contrast, light PA (day/week) showed no significant difference between inactive and active participants in the pre-COVID-19 pandemic group (*p* = 0.337).

The result indicated a significant difference in the amount of PA since the implementation of COVID-19 restrictions. The participants reported declined PA as 8.3% and 11.8% of the total inactive students (*n* = 313) became “much more” and “more” inactive, while a 0.0% and 26.5% of the total active students (*n* = 98) became “much more” and “more active”. Hence, due to the COVID-19 pandemic, most of students became more inactive since the implementation of COVID-19 restrictions.

The majority of the students from both groups participated in jogging; 80.1% of the total inactive group and 83.6% of the total active group, respectively. The type of activities among the “inactive” and “active” groups was reported to be “not so similar” for the inactive group (29.7%) and “very similar” for the active group (33.7%) compared to before restrictions were put in place (76.9% in the inactive and 23.1% in the active group).

### 3.2. Indoor and Outdoor Physical Activity Behaviour in Active and Inactive Groups

Table 3 demonstrates that most inactive and active students participated in PA in “both outdoor and indoor” environments: In outdoor environments, 71.4% of inactive students performed their outdoor PA in an open environment, such as in the school field, compared to 28.6% of the active students, who participated in the indoor environment of their residential location or surroundings. However, the PA location showed no significant difference among indoor, outdoor, and none in the pre-COVID-19 pandemic group (*p* = 0.373).

The active students spent significantly more time and days/week on the three types of physical activities: vigorous, moderate, and light, compared to inactive students (*p* < 0.001, *p* = 0.042, and *p* < 0.001), respectively.

### 3.3. The Nature and Place of Physical Activity Engagement during the COVID-19 Pandemic

Table 4 reveals that there was a significant difference between “more active”, “same”, and “less active” in the engagement of “moderate–vigorous” PA (*p* < 0.001). The participants’ responses were divided into three Likert scales of “more active”, “same”, and “less active”, with the total number of respondents in each group recorded as 96, 85, and 230, respectively. Regarding the nature of the PA performed, there was a significant difference between “more active”, “same–active”, and “less active” in response to the variables “I enjoy digging in the earth and getting dirt on my hands” and “I take notice of wildlife wherever I am” (*p* = 0.034 and 0.001, respectively). On the other hand, during the pre-pandemic period, there was a significant difference between “more active”, “the same”, and “less active” in terms of moderate-to-vigorous activities (*p* < 0.001).

Five of the assessing variables showed a significant association between “more active” and “less active” on the barriers and facilitators associated with the place of PA engagement: The variables are “How enjoyable is it for you to exercise regularly right now?”, “How difficult is it for you to exercise regularly right now?”, “How detailed of a plan do you have for exercising regularly right now?”, “How confident are you that you can exercise regularly right now?”, and “Have you found it more challenging to engage in regular exercise since the onset of social distance?” (*p* < 0.001, respectively).

Regarding the participant’s responses to their well-being during the COVID-19 pandemic, Table 5 shows there was a significant association between the variables “maintaining close relationships has been difficult” and “it is difficult to voice their opinions on controversial matters” (*p* < 0.001, respectively). Similarly, during the pre-pandemic period, these variables showed significant differences among “more active”, “the same”, and “less active” (*p* < 0.001).

## 4. Discussion

This study aimed to analyze the PA and well-being of dental students from different countries during the pandemic. The obtained research results turned out to be interesting from a public health point of view. Considering the impact of the lockdown during the pandemic period, the findings were compared with the pre-pandemic period. The global transmission of COVID-19 prompted the authorities of the world to apply unprecedented containment measures. Quarantine upset the normality of students’ daily life, forcing social distancing and self-isolation. The results from this study revealed a significant rise in the level of inactivity between the two groups, “inactive” and “active group”. The percentage of inactive students becoming less physically active increased compared to the active students becoming less physically active since the implementation of COVID-19 restrictions. Therefore, home workouts and PA remained the only option to stay active during the COVID-19 pandemic. Public health emergencies might have a psychological impact on students’ health, expressed as changes in activity location, types, and increased stress amongst students.

To the best of our knowledge, this is the first multinational study that was carried out to measure the impact of the COVID-19 pandemic on the PA behavior and well-being of dental students in the studied countries. The current study showed that most of the students were active prior to the COVID-19 pandemic. Similarly, high levels of PA were found among (*n* = 1572) participants in Brazil prior to the COVID-19 pandemic, with 83% of males and 85% of females exceeding 150 min per week of moderate-to-vigorous activities [36]. Our study also demonstrated that during the COVID-19 pandemic, there was a significant decrease in PA, and the students became less active in both groups. It also indicated that inactive students became less active than active students. These findings are similar to a study conducted among Italian subjects that investigated the effect of COVID-19 on PA level, indicating that the effect of COVID-19 significantly reduced PA engagement during the COVID-19 pandemic [29]. This result was also similar to the findings of Lesser et al. [30]. Anxiety and lack of motivation could be factors that can decrease and change all types of students’ PA [37]. Cornine [38] reported that the fear and anxiety of students about COVID-19 could be related to the effect of its restrictions on their PA behavior, consequently affecting their mood and making them inactive. Public health restrictions can influence PA behavior at several levels.

A dramatic change in daily schedules and preferences may be attributed to COVID-19 restrictions, as low-intensity tasks such as housework (cooking, washing dishes, or gardening) as well as staying at home and spending time on sedentary pursuits can be factors that make students inactive [39,40,41].

In the inactive population, there were significant differences in well-being outcomes between those who were more active, the same, or less active, but not in the active population. Inactive individuals’ well-being was strongly linked to PA, and PA was significantly reduced during the COVID-19 pandemic [30].

The results showed that most inactive and active students participated in PA both outdoors and indoors, and the active students spent significantly more days/week and min/each day than the inactive students performing the three types of physical activities: vigorous, moderate, and light. Colley and Bushnik [42] Stated that people had good or excellent mental well-being and experienced excellent general health and comfort if they exercised outdoors compared with those who did not. Therefore, most students reported that the environment is crucial and essential to their PA behavior, especially if it is natural. Nature exposure has been linked to better mental health, with perceived stress behaving as a mediator [43,44]. Lack of access to regular PA posed challenges to the immune system and physical health, exacerbating pre-existing diseases associated with sedentary lifestyles. Furthermore, a lack of access to exercise and PA had exacerbated the stress and/or anxiety that many felt due to their isolation from a normal social life.

Ab initio, the participants reported that there was a difference in PA engagement (moderate–vigorous) among “more active”, “same”, and “less active”. The “more active” students spent significantly more minutes each day compared to their “same” and “less active” counterparts since the implementation of COVID-19 restrictions. This might be due to the effect of these COVID-19 measures on the routine of daily student life and anxiety during the quarantine [14,45,46].

Although this study indicated that students engage in indoor and outdoor activities, a higher proportion participated in indoor PA during the COVID-19 restriction. This is in agreement with the finding of a previous study [47]. Usually, the individual chooses whether to perform indoor or outdoor PA, but the spread of COVID-19 hampered this decision, leading to the closure of sporting and recreation centers such as stadiums, gyms, and physiotherapy centers, thus compelling the students to engage in indoor PA [29]. These factors can serve as a barrier to PA engagement in natural environments. The current study showed that some participants enjoyed being exposed to nature and the outdoors, while others reported they enjoyed being indoors. The inability to perform exercise and PA experienced by many students has been found to create a psychosocial imbalance in mental health due to isolation and being denied their usual social activities [48].

The majority of the students in our study reported not being satisfied as they have been denied their normal daily activities and connection with nature (cannot enjoy outdoor events and wildlife events). The majority of the students described their “well-being state” during the period as being restless, frustrated, having trouble relaxing, nervous/worrying, and most especially feeling afraid. They also reported their “current feeling” as being denied support from nature and decreasing the available opportunities to explore nature. Due to this restraint on the daily events of the students, they were less happy, felt psychosocially stressed, and could not perform outdoor exercise, which is considered a healthy living condition.

This study revealed significant differences between the ”more active”, ”same”, and ”less active” groups on students’ well-being during the COVID-19 pandemic. This showed that the restriction imposed on the students due to the pandemic depicted a positive correlation to their quality of life [49,50]. The psychological impacts and the reduction in the students’ physical activities could be due to the quarantine at home, increasing their sedentary behavior and decreasing motivation status. This is characterized by the increased use of mobile devices and increase in average time spent watching television [51]. Social supports, confidence, and the opportunities to engage in PA are essential to maintaining a healthy lifestyle and reducing all-cause mortality [52].

Finally, recruiting the participants to answer the online questionnaire via a link shared using personal WhatsApp, Facebook Messenger, and Twitter social media might result in some selection bias. The study’s ability to investigate changes in well-being owing to the implementation of public health policies was hampered by the inability to assess pre-COVID-19 anxiety and well-being states. Furthermore, pre-COVID-19 PA behavior assessments would supplement the subjective assessment of changes in the level of PA. The different seasonal changes and the magnitude of COVID-19 public health reactions in the selected nations could influence collective chances for outdoor and indoor PA, which might lead to some selection bias.

## 5. Conclusions

COVID-19 created a compulsory shutdown of almost all routine human activities, forcefully restraining students from leaving their homes unless emergency needs arise. These regulations affected students’ PA and well-being, particularly the psychosocial and boredom-induced stress that resulted from reduced PA. Because the long-term impacts of the COVID-19 pandemic are uncertain, more research is solicited to further investigate the long-term impact of COVID-19 on PA, mental health, and quality of life. We recommended that students stay fit and active even with COVID-19 pandemic restrictions by engaging in indoor PA to benefit from the positive effect of PA while mitigating the risk of COVID-19 transmission.

## Figures and Tables

**Table 1:** Participants’ demographic description.

Characteristics	Pre-Pandemic	During Pandemic
	*n* (%)	*n* (%)
Age		
16–20	18 (18.4)	141 (34.35)
21–25	38 (38.8)	139 (33.82)
26–29	38 (38.8)	115 (27.98)
30–40	4 (4.1)	16 (3.89)
Country		
Cambodia	16 (16.3)	56 (13.63)
India	43 (43.9)	189 (45.99)
Malaysia	16 (16.3)	46 (11.19)
Saudi Arabia	23 (23.5)	120 (29.20)
Sex		
Male	53 (54.1)	216 (52.55)
Female	45 (45.9)	195 (47.45)

**Table 2:** Physical activity engagement, facilitators, barriers, and change in type of activities since COVID-19.

Participant Characteristic	Pre-COVID-19 Pandemic			During COVID-19 Pandemic		
	Inactive *n* (%)	Active *n* (%)	*p*-Value	Inactive *n* (%)	Active *n* (%)	*p*-Value
Overall ^c^	26 (27)	72 (73)		313 (76)	98 (24)	
India ^c^	13 (30)	30 (70)		145 (46.3)	44 (44.9)	
Cambodia ^c^	5 (31)	11 (69)		88 (28.1)	32 (32.7)	
Saudi Arabia ^c^	8 (35)	15 (65)		46 (14.7)	10 (10.2)	
Malaysia ^c^	0 (0)	16 (100)		34 (10.9)	12 (12.2)	
Godin Leisure Score (mean, SD) ^d^	120.00 ± 0.000	74.51 ± 35.890	0.001	30.89 ± 25.353	42.96 ± 23.216	0.001 * ^a^
PA engagement ^d^						
Vigorous PA (min/each day)	5.00 ± 0.000	3.56 ± 1.803	0.001 * ^a^	27.78 ± 27.717	44.08 ± 35.314	0.001 * ^a^
Vigorous PA (day/week)	48.42 ± 25.661	53.86 + 33.949	0.515 ^a^	2.19 ± 1.933	3.03 ± 1.992	0.001 * ^a^
Moderate PA (min/each day)	4.00 ± 0.000	3.84 ± 0.406	0.001 * ^a^	27.09 ± 20.072	33.88 ± 26.287	0.007 * ^a^
Moderate PA (day/week)	58.52 + 25.661	54.94 ± 32.448	0.417 ^a^	2.58 ± 2.191	3.08 ± 1.887	0.042 * ^a^
Light PA (min/each day)	5.00 ± 0.000	3.84 ± 0.406	0.001 * ^a^	22.86 ± 24.161	50.51 ± 63.900	0.001 * ^a^
Light PA (day/week)	48.42 ± 25.661	40.63 ± 32.820	0.337 ^a^	3.37 ± 2.612	3.74 ± 2.421	0.213
Changes in PA						
Much more	0 (0)	0 (0)		26 (8.3)	0 (0.0)	
More	5 (23)	17 (77)	0.001 * ^b^	37 (11.8)	26 (26.5)	0.001 * ^b^
About the same	0 (0)	14 (100)		51 (16.3)	30 (30.6)	
Less	8 (19)	34 (81)		113 (36.1)	30 (30.6)	
Much less	13 (65)	7 (35)		86 (27.5)	12 (12.2)	
The most common type of physical activity						
Jogging	13 (33)	26 (67)		253 (80.1)	82 (83.6)	
Games	0 (0)	2 (100)	0.056 ^b^	12 (3.8)	4 (4.1)	
Yoga	1 (5)	20 (95)		12 (3.8)	4 (4.1)	0.564 ^b^
Gym	5 (25)	15 (75)		28 (8.9)	4 (4.1)	
None	7 (44)	9 (56)		8 (2.6)	4 (4.1)	
Change in type of activity						
Extremely similar	13 (38)	21 (62)		18 (5.8)	3 (3.1)	
Very similar	0 (0)	22 (100)	0.004 ^b^	88 (28.1)	33 (33.7)	
Somewhat similar	8 (27)	22 (73)		76 (24.3)	21 (21.4)	0.698 ^b^
Not so similar	1 (17)	5 (83)		93 (29.7)	28 (28.6)	
Not at all similar	4 (67)	2 (33)		38 (12.1)	13 (13.3)	
Most common physical activity location ^c^						
Within the dormitory	8 (29)	20 (71)		114 (36.4)	37 (24.5)	
School field and track	13 (22)	45 (78)	0.373 ^b^	191 (61.0)	57 (23.0)	0.693 ^b^
None	5 (42)	7 (58)		8 (66.7)	4 (2.6)	

* Significance difference (*p* < 0.05), ^a^ independent t-test, ^b^ chi-square test, ^c^ data expressed as number and percentage *n* (%), ^d^ data expressed in mean ± S.D.

**Table 3:** Summaries of the results of indoor and outdoor physical activity behavior in active and inactive students.

Participant Characteristic	Pre-COVID-19 Pandemic			During COVID-19 Pandemic		
	Inactive *n* (%) 26 (27)	Active *n* (%) 72 (73)	*p*-Value	Inactive *n* (%) 313 (76)	Active *n* (%) 98 (24)	*p*-Value
PA location ^b^						
Indoor	8 (29)	20 (71)		157 (84.9)	28 (15.1)	
Outdoor	13 (22)	45 (78)	0.373 ^b^	46 (63.9)	26 (36.1)	0.001 * ^b^
Both	5 (42)	7 (58)		110 (71.4)	44 (28.6)	
Is it a natural environment? *^c^*						
Yes	20 (29)	50 (71)	0.469 ^b^	255 (73.9)	90 (26.1)	0.015 * ^b^
No	6 (21)	22 (79)		58 (87.9)	8 (12.1)	
Is the environment necessary in your chosen PA? ^c^						
Extremely important	13 (59)	9 (41)		71 (71.7)	28 (28.3)	
Very important	0 (0)	16 (100)		149 (77.2)	44 (22.8)	
Somewhat important	8 (19)	34 (81)	0.001 * ^b^	56 (81.2)	13 (18.8)	0.174 ^b^
Not so important	0 (0)	10 (100)		28 (68.3)	13 (31.7)	
Not all important	5 (63)	3 (37)		9 (100.0)	0 (0.0)	
How essential is nature in your chosen PA? ^c^						
Extremely important	13 (33)	27 (67)		67 (70.5)	28 (29.5)	
Very important	0 (0)	16 (100)	0.081 ^b^	143 (77.7)	41 (22.3)	0.086 ^b^
Somewhat important	8 (30)	19 (70)		69 (81.2)	16 (18.8)	
Not so important	5 (39)	8 (61)		25 (65.8)	13 (34.2)	
Not all important	26 (27)	72 (73)		9 (100.0)	0 (0.0)	

* Significance difference (*p* < 0.05), ^b^ chi-square test, ^c^ data expressed as number and percentage *n (%).*

**Table 4:** The participant’s responses to the nature and place of physical activity engagement during the COVID-19 pandemic.

Participant Characteristic	Pre-COVID-19 Pandemic				During COVID-19 Pandemic			
	More Active *n* = 17	The Same *n* = 69	Less Active *n* = 12	*p*-Value	More Active *n* = 96	The Same *n* = 85	Less Active *n* = 230	*p*-Value
Connectedness to nature on scale number ^d^	4.24 ± 2.251	3.93 ± 2.952	3.08 ± 3.502	0.560 ^c^	6.13 ± 2.079	5.74 ± 2.376	5.91 ± 2.561	0.562 ^c^
Type of the PA (moderate–vigorous activity minutes each day) ^d^	98.53 ± 18.689	58.93 ± 36.427	26.25 ± 22.676	0.001 * ^c^	55.83 ± 39.187	49.24 ± 37.718	34.43 ± 25.241	0.001 ^c^
Are you engaging in indoor or outdoor PA ^e^								
Indoor	0 (0)	11 (79)	3 (21)		33 (17.8%)	24 (13.0%)	128 (69.2)	
Outdoor	14 (21)	54 (79)	0 (0)	0.001 * ^b^	19 (26.4%)	17 (23.6%)	36 (50.6%)	0.001 * ^b^
Both	3 (19)	4 (25)	9 (56)		44 (28.6%)	44 (28.6%)	66 (42.9)	
Nature of Activity ^d^								
I enjoy being outdoors, even in unpleasant weather	2.18 ± 0.529	2.22 ± 1.110	2.00 ± 0.739	0.784	2.54 ± 1.123	2.66 ± 1.086	2.40 ± 1.162	0.175 ^c^
My ideal vacation spot would be a remote wideness area	2.29 ± 0.686	2.68 ± 1.194	2.25 ± 0.866	0.247	2.75 ± 0.973	2.88 + 1.248	3.03 ± 1.186	0.131 ^c^
I enjoy digging in the earth and getting dirt on my hands	2.12 ± 0.697	2.72 ± 0.856	2.08 ± 0.793	0.004	2.50 + 0.918	2.13 + 1.252	2.45 ± 1.059	0.034 * ^c^
I take notice of wildlife wherever I am	3.88 ± 0.781	3.54 ± 0.948	3.67 ± 0.985	0.381	3.63 + 0.861	3.12 ± 1.304	3.61 ± 1.046	0.001 * ^c^
I don’t often go out in nature	2.00 ± 0.000	2.23 ± 0.825	2.50 ± 1.168	0.258	2.63 + 0.954	2.49 ± 1.377	2.48 ± 1.077	0.564 ^c^
The thought of being deep in the woods away from civilization is frightening	2.88 ± 0.928	2.14 ± 0.670	2.17 ± 0.389	0.001	2.79 + 1.004	2.84 ± 1.404	2.71 ± 1.108	0.666 ^c^
Barriers and Facilitators to PA Engagement ^d^								
How beneficial is it for you to exercise regularly right now?	3.12 ± 0.45	3.68 ± 0.86	3.33 ± 1.21	0.042 ^c^	4.00 ± 1.046	4.00 ± 816	3.83 ± 1.057	0.259 ^c^
How enjoyable is it for you to exercise regularly right now?	3.94 ± 0.49	3.33 ± 0.53	3.17 ± 1.27	0.001 ^c^	3.71 ± 0.983	3.53 ± 1.097	3.24 ± 1.141	0.001 ^c^
How difficult is it for you to exercise regularly right now?	1.35 ± 0.82	2.10 ± 1.22	3.42 ± 1.04	0.001 ^c^	2.61 ± 0.925	2.24 ± 1.192	3.04 ± 1.251	0.001 ^c^
How confident are you that you can exercise regularly right now?	3.71 ± 0.58	3.59 ± 0.80	2.67 ± 0.78	0.001 ^c^	3.50 ± 962	3.58 ± 1.051	3.03 ± 1.051	0.001 ^c^
How motivated are you to exercise regularly right now?	3.18 ± 0.59	3.21 ± 0.83	2.75 ± 1.18	0.242 ^c^	3.75 + 0.929	3.53 ± 0.959	2.86 + 1.078	0.001 ^c^
How detailed of a plan do you have for exercising regularly right now?	2.94 ± 1.029	2.88 ± 1.092	2.67 ± 1.155	0.779 ^c^	3.00 + 1.086	2.92 ± 1.071	2.69 ± 1.085	0.435 ^c^
Have you found it more challenging to engage in regular exercise since the onset of social distance?	2.82 ± 1.27	3.13 ± 1.39	2.83 ± 1.27	0.587 ^c^	2.42 + 1.002	2.41 ± 1.228	3.20 ± 1.280	0.001 * ^c^
Current Feeling about Exercise ^d^								
How many opportunities do you have for exercising regularly right now?	3.82 ± 0.529	3.38 ± 0.806	2.83 ± 0.718	0.001 * ^c^	3.25 ± 0.929	3.09 ± 0.868	2.84 ± 0.783	0.001 * ^c^
How much support do you have for exercising regularly right now?	1.29 ± 0.686	2.03 ± 1.000	2.52 ± 0.669	0.003 * ^c^	2.88 ± 0.976	2.53 ±1.097	2.49 ± 0.998	0.007 * ^c^

* Significance difference (*p* < 0.05), ^b^ chi-square test, ^c^ one-way ANOVA test, ^d^ data expressed in mean ± S.D. ^e^ data expressed as number and percentage *n* (%).

**Table 5:** The participant’s responses to questions on their well-being during the COVID-19 pandemic.

Participant Characteristic	Pre-COVID-19 Pandemic				During COVID-19 Pandemic			
	More Active *n* = 17	The Same *n* = 69	Less Active *n* = 12	*p*-Value	More Active *n* = 96	The Same *n* = 85	Less Active *n* = 230	*p*-Value
Well-being Outcome ^d^								
Being so restless that it is hard to sit still	3.65 ± 0.786	3.16 ± 1.184	2.25 ± 0.965	0.005 * ^c^	1.83 ± 0.627	1.89 ± 0.756	2.08 ± 0.900	0.022 * ^c^
Feeling nervous, anxious, or on edge	1.12 ± 0.332	1.70 ± 0.626	2.50 ± 0.674	0.001 * ^c^	1.83 ± 0.749	1.72 ± 0.766	2.16 ± 0.828	0.001 * ^c^
Not being able to stop or control worrying	1.18 ± 0.529	1.62 ± 0.712	1.65 ± 0.801	0.001 * ^c^	1.75 ± 0.847	2.10 ± 1.044	2.11 ± 0.956	0.005 * ^c^
Worrying too	1.17 ± 0.429	1.58 ± 0.864	2.58 ± 0.900	0.001 * ^c^	1.75 ± 0.834	2.09 ± 0.019	2.11 ± 0.955	0.066 ^c^
Trouble relaxing much about different things	2.00 ± 0.000	1.96 ± 0.605	2.25 ± 0.866	0.001 * ^c^	1.67 ± 0.691	1.94 ± 0.729	1.98 ± 0.816	0.001 * ^c^
Feeling afraid, as if something awful might happen	1.71 ± 0.686	1.90 ± 0.860	2.25 ± 0.866	0.288 ^c^	1.83 ± 0.749	2.19 ± 1.732	2.04 ± 0.717	0.017 * ^c^
Becoming easily annoyed or irritable about different things	2.06 ± 0.243	1.94 ± 0.511	2.08 ± 0.669	0.514 ^c^	1.88 ± 0.785	2.19 ± 0.732	2.04 ± 0.717	0.081 ^c^
Most people see me as loving and affectionate	5.06 ± 1.600	5.61 ± 1.526	4.75 ± 1.658	0.131 ^c^	5.21 ± 1.641	5.07 ± 1.631	5.44 ± 1.598	0.157 ^c^
Maintaining close relationships has been difficult	1.18 ± 0.529	3.14 ± 2.158	4.75 ± 1.658	0.001 * ^c^	2.92 ± 1.715	3.26 ± 2.065	4.03 ± 1.964	0.001 * ^c^
I often feel lonely because I have few close friends with whom to share my concerns	2.00 ± 0.354	2.74 ± 1.876	2.92 ± 1.084	0.208 ^c^	3.00 ± 1.667	2.79 ± 1.928	3.49 ± 1.960	0.006 * ^c^
This is frustrating for me as I enjoy personal and mutual conversation with family members and friends	4.41 ± 0.618	4.13 ± 2.255	4.67 ± 1.923	0.655 ^c^	3.88 ± 1.932	3.51 ± 2.223	4.26 ± 2.121	0.015 * ^c^
I have not experienced many warm and trusting relationships with others	1.35 ± 1.222	2.62 ± 1.791	3.58 ± 1.975	0.003 * ^c^	2.63 ± 1.687	2.98 ± 1.812	3.17 ± 1.948	0.053 * ^c^
It is difficult for me to voice my opinions on controversial matters.	1.29 ± 0.985	2.19 ± 1.801	3.58 ± 1.881	0.003 * ^c^	2.13 ± 1.401	1.94 ± 1.815	2.90 ± 1.767	0.001 * ^c^

* Significance difference (*p* < 0.05), ^c^ one-way ANOVA test, ^d^ data expressed as number and percentage *n* (%).

## Data Availability

All relevant data related to this study have been provided in this article.
